# Supplementary material for: A Humanized Yeast Phenomic Model of Deoxycytidine Kinase to Predict Genetic Buffering of Nucleoside Analog Cytotoxicity
Source: Genes (Basel). 2019 Sep 30;10(10):770. doi: 10.3390/genes10100770 (PMC6826991; doi:10.3390/genes10100770)
Supplement: Supplementary file 1 [file genes-10-00770-s001.zip › Additional_Files/Additional_File7_GOTermHeatmaps.zip!/Additional_File7_GOTermHeatmaps/vacuole_organization.pdf]

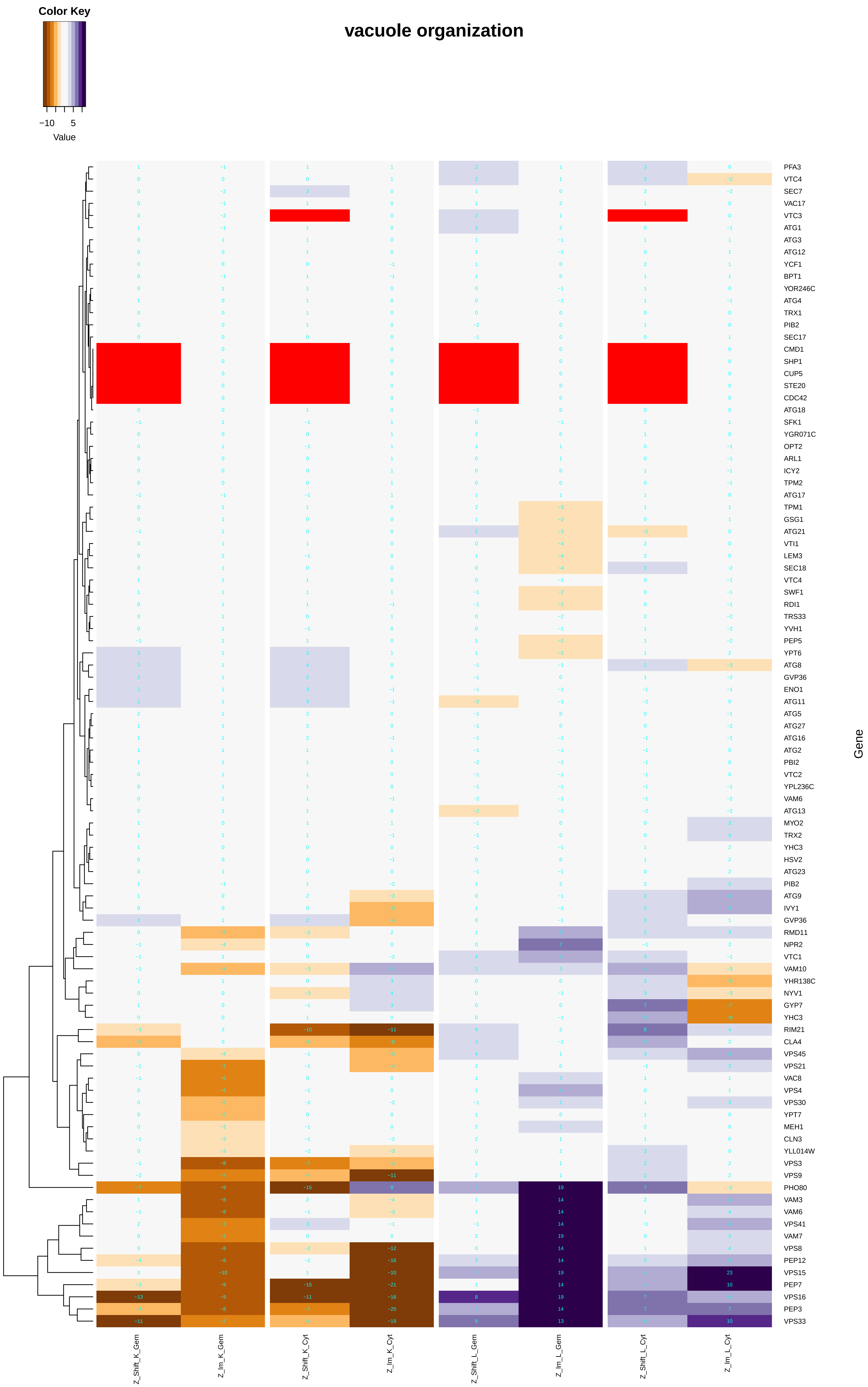
Color Key
−10
5
Value
vacuole organization
PFA3
VTC4
SEC7
VAC17
VTC3
ATG1
ATG3
ATG12
YCF1
BPT1
YOR246C
ATG4
TRX1
PIB2
SEC17
CMD1
SHP1
CUP5
STE20
CDC42
ATG18
SFK1
YGR071C
OPT2
ARL1
ICY2
TPM2
ATG17
TPM1
GSG1
ATG21
VTI1
LEM3
SEC18
VTC4
SWF1
RDI1
TRS33
YVH1
PEP5
YPT6
ATG8
GVP36
ENO1
ATG11
ATG5
ATG27
ATG16
ATG2
PBI2
VTC2
YPL236C
VAM6
ATG13
MYO2
TRX2
YHC3
HSV2
ATG23
PIB2
ATG9
IVY1
GVP36
RMD11
NPR2
VTC1
VAM10
YHR138C
NYV1
GYP7
YHC3
RIM21
CLA4
VPS45
VPS21
VAC8
VPS4
VPS30
YPT7
MEH1
CLN3
YLL014W
VPS3
VPS9
PHO80
VAM3
VAM6
VPS41
VAM7
VPS8
PEP12
VPS15
PEP7
VPS16
PEP3
VPS33
Gene
Z_Shift_K_Gem
Z_Im_K_Gem
Z_Shift_K_Cyt
Z_Im_K_Cyt
Z_Shift_L_Gem
Z_Im_L_Gem
Z_Shift_L_Cyt
Z_Im_L_Cyt

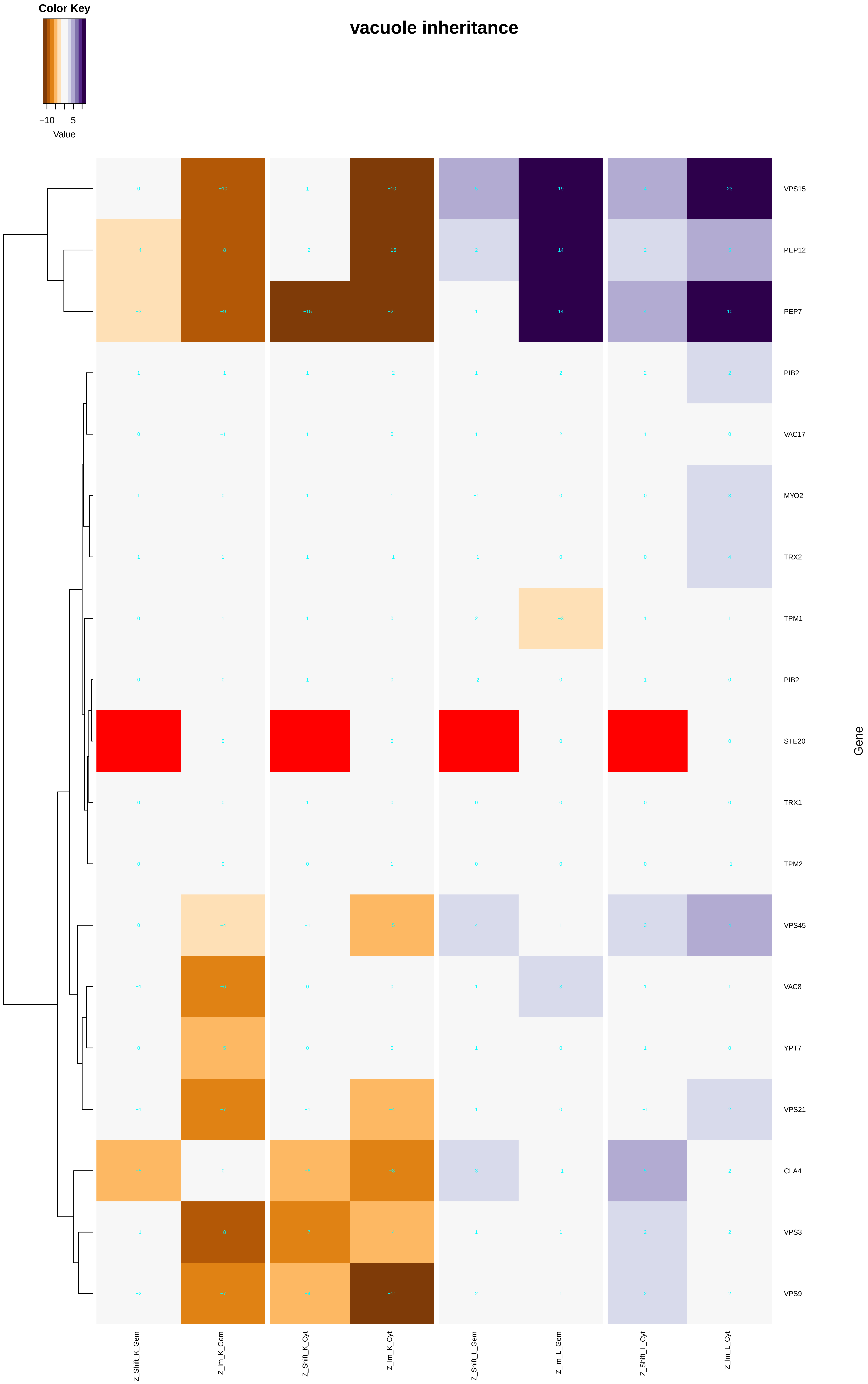

Color Key
−10
5
Value
vacuole inheritance
VPS15
PEP12
PEP7
PIB2
VAC17
MYO2
TRX2
TPM1
PIB2
STE20
TRX1
TPM2
VPS45
VAC8
YPT7
VPS21
CLA4
VPS3
VPS9
Gene
Z_Shift_K_Gem
Z_Im_K_Gem
Z_Shift_K_Cyt
Z_Im_K_Cyt
Z_Shift_L_Gem
Z_Im_L_Gem
Z_Shift_L_Cyt
Z_Im_L_Cyt

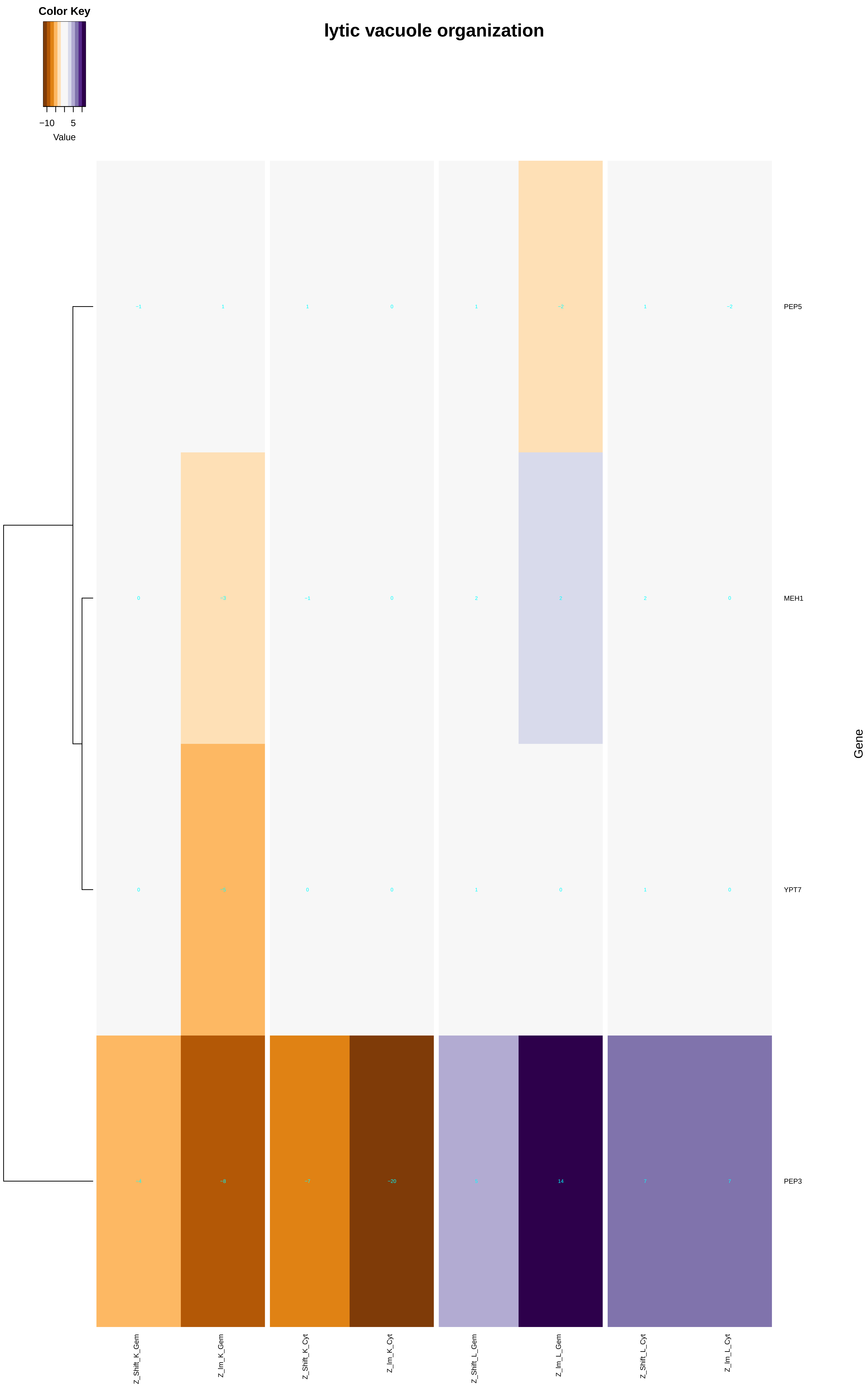

lytic vacuole organization
Color Key
−10
5
Value
Gene
PEP5
MEH1
YPT7
PEP3
Z_Shift_K_Gem
Z_Im_K_Gem
Z_Shift_K_Cyt
Z_Im_K_Cyt
Z_Shift_L_Gem
Z_Im_L_Gem
Z_Shift_L_Cyt
Z_Im_L_Cyt
−1
1
1
0
1
−2
1
−2
0
−3
−1
0
2
2
2
0
0
0
0
1
0
1
0
−8
−7
−20
14
7
7

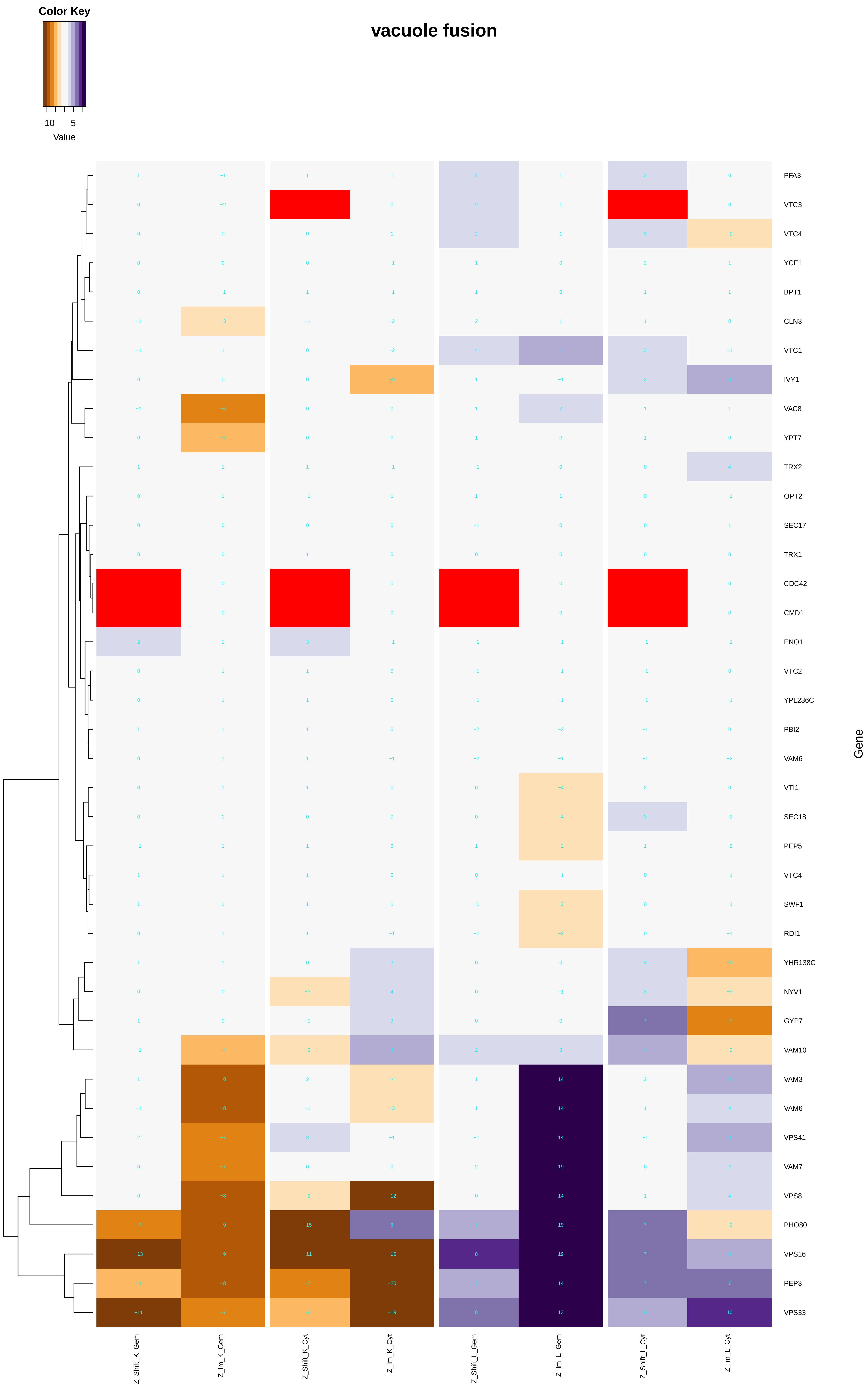
vacuole fusion
Color Key
−10
5
Value
PFA3
VTC3
VTC4
YCF1
BPT1
CLN3
VTC1
IVY1
VAC8
YPT7
TRX2
OPT2
SEC17
TRX1
CDC42
CMD1
ENO1
VTC2
YPL236C
PBI2
VAM6
VTI1
SEC18
PEP5
VTC4
SWF1
RDI1
YHR138C
NYV1
GYP7
VAM10
VAM3
VAM6
VPS41
VAM7
VPS8
PHO80
VPS16
PEP3
VPS33
Gene
Z_Shift_K_Gem
Z_Im_K_Gem
Z_Shift_K_Cyt
Z_Im_K_Cyt
Z_Shift_L_Gem
Z_Im_L_Gem
Z_Shift_L_Cyt
Z_Im_L_Cyt

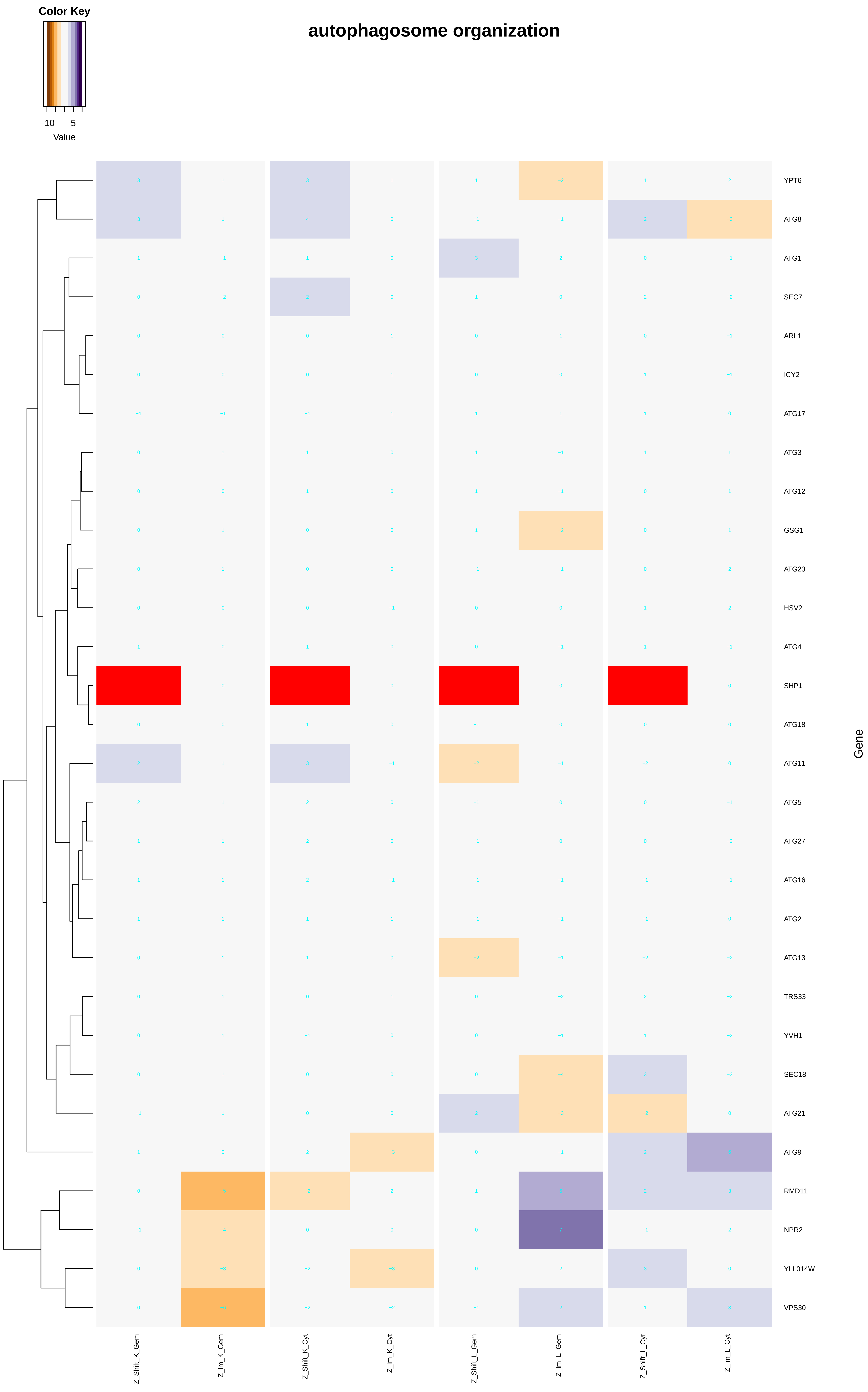
Color Key
−10
5
Value
autophagosome organization
YPT6
ATG8
ATG1
SEC7
ARL1
ICY2
ATG17
ATG3
ATG12
GSG1
ATG23
HSV2
ATG4
SHP1
ATG18
ATG11
ATG5
ATG27
ATG16
ATG2
ATG13
TRS33
YVH1
SEC18
ATG21
ATG9
RMD11
NPR2
YLL014W
VPS30
Gene
Z_Shift_K_Gem
Z_Im_K_Gem
Z_Shift_K_Cyt
Z_Im_K_Cyt
Z_Shift_L_Gem
Z_Im_L_Gem
Z_Shift_L_Cyt
Z_Im_L_Cyt

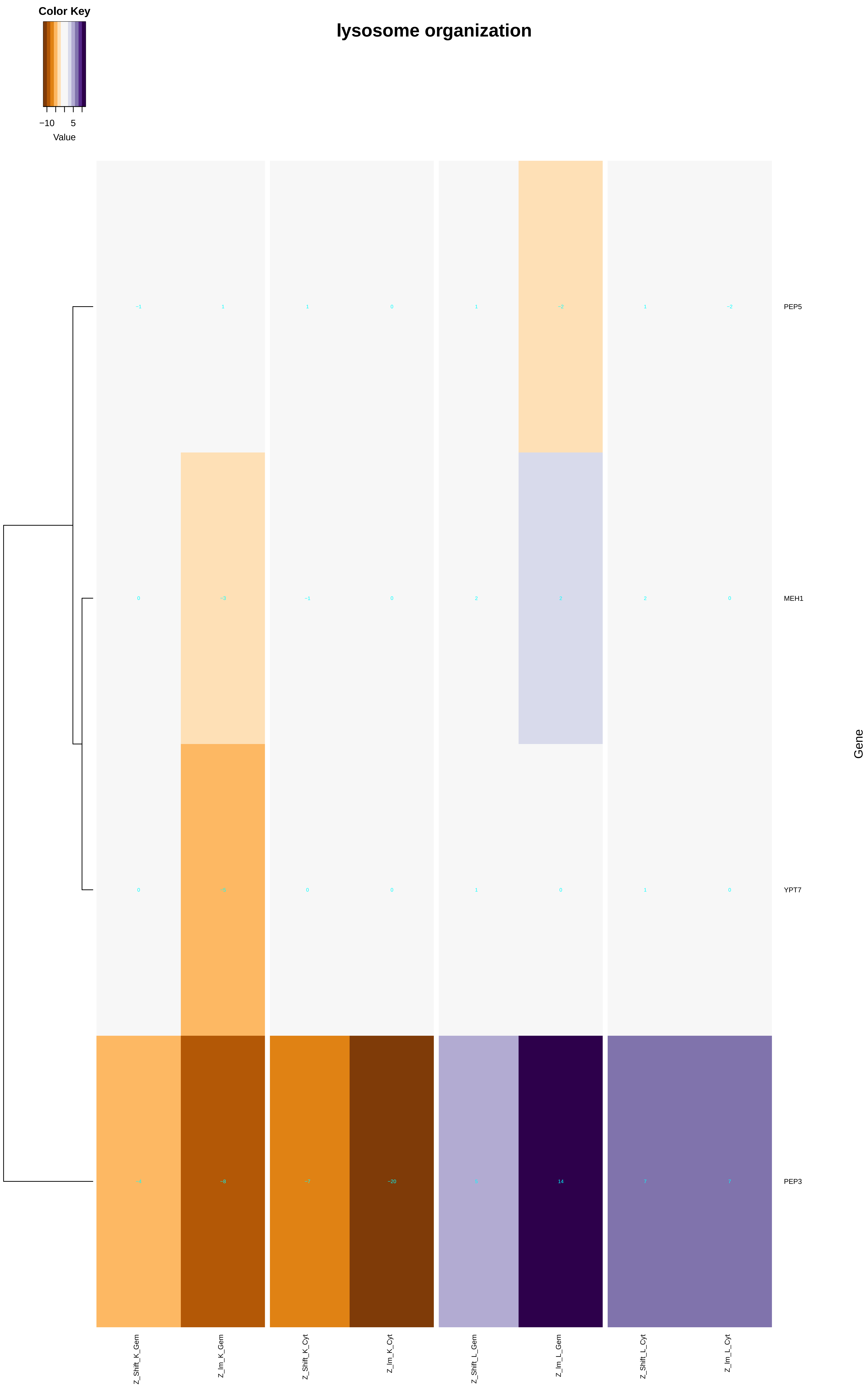
lysosome organization
Color Key
−10
5
Value
PEP5
MEH1
YPT7
PEP3
Gene
Z_Shift_K_Gem
Z_Im_K_Gem
Z_Shift_K_Cyt
Z_Im_K_Cyt
Z_Shift_L_Gem
Z_Im_L_Gem
Z_Shift_L_Cyt
Z_Im_L_Cyt

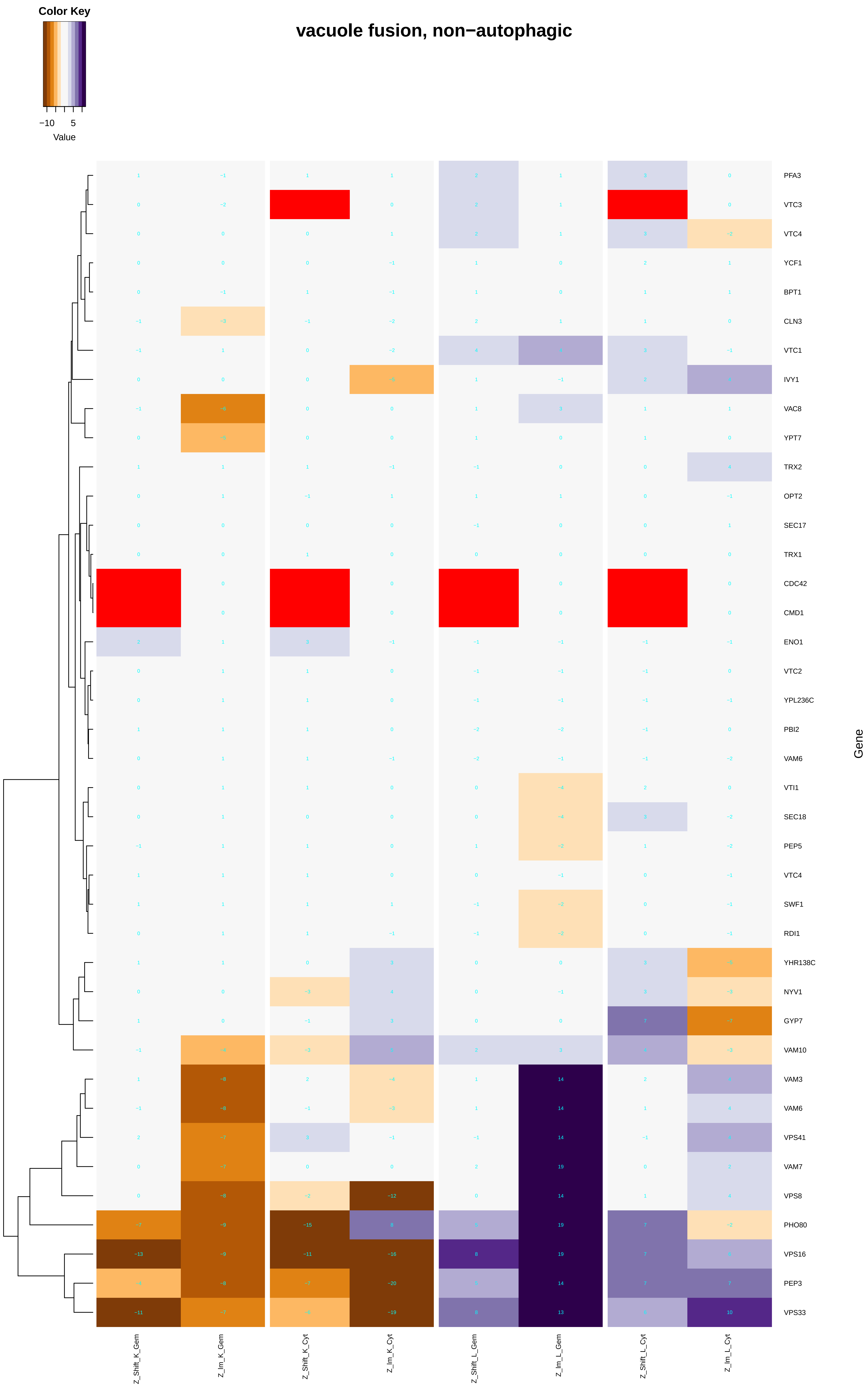
Color Key
−10
5
Value
vacuole fusion, non−autophagic
PFA3
VTC3
VTC4
YCF1
BPT1
CLN3
VTC1
IVY1
VAC8
YPT7
TRX2
OPT2
SEC17
TRX1
CDC42
CMD1
ENO1
VTC2
YPL236C
PBI2
VAM6
VTI1
SEC18
PEP5
VTC4
SWF1
RDI1
YHR138C
NYV1
GYP7
VAM10
VAM3
VAM6
VPS41
VAM7
VPS8
PHO80
VPS16
PEP3
VPS33
Gene
Z_Shift_K_Gem
Z_Im_K_Gem
Z_Shift_K_Cyt
Z_Im_K_Cyt
Z_Shift_L_Gem
Z_Im_L_Gem
Z_Shift_L_Cyt
Z_Im_L_Cyt

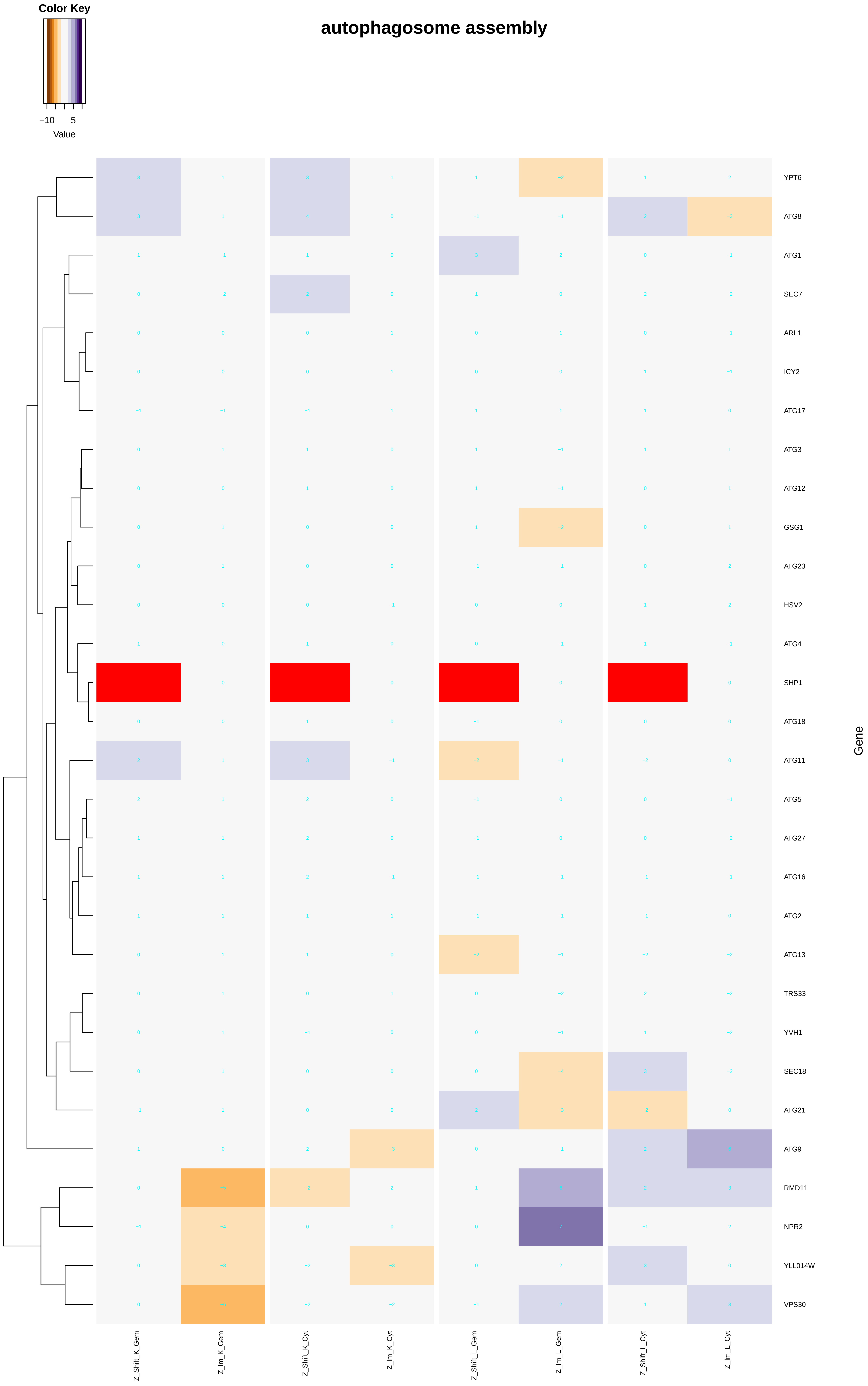
Color Key
−10
5
Value
autophagosome assembly
YPT6
ATG8
ATG1
SEC7
ARL1
ICY2
ATG17
ATG3
ATG12
GSG1
ATG23
HSV2
ATG4
SHP1
ATG18
ATG11
ATG5
ATG27
ATG16
ATG2
ATG13
TRS33
YVH1
SEC18
ATG21
ATG9
RMD11
NPR2
YLL014W
VPS30
Gene
Z_Shift_K_Gem
Z_Im_K_Gem
Z_Shift_K_Cyt
Z_Im_K_Cyt
Z_Shift_L_Gem
Z_Im_L_Gem
Z_Shift_L_Cyt
Z_Im_L_Cyt
